# 3D bioprinted scaffolds for diabetic wound-healing applications

**DOI:** 10.1007/s13346-022-01115-8

**Published:** 2022-01-11

**Authors:** Katie Glover, Essyrose Mathew, Giulia Pitzanti, Erin Magee, Dimitrios A. Lamprou

**Affiliations:** School of Pharmacy, Queen’s University Belfast, 97 Lisburn Road, Belfast, BT9 7BL UK

**Keywords:** Diabetic foot ulcer, Diabetes mellitus, Bioprinting, Wound healing, Drug delivery, Levofloxacin

## Abstract

The treatment strategy required for the effective healing of diabetic foot ulcer (DFU) is a complex process that is requiring several combined therapeutic approaches. As a result, there is a significant clinical and economic burden associated in treating DFU. Furthermore, these treatments are often unsuccessful, commonly resulting in lower-limb amputation. The use of drug-loaded scaffolds to treat DFU has previously been investigated using electrospinning and fused deposition modelling (FDM) 3D printing techniques; however, the rapidly evolving field of bioprinting is creating new opportunities for innovation within this research area. In this study, 3D-bioprinted scaffolds with different designs have been fabricated for the delivery of an antibiotic (levoflocixin) to DFU. The scaffolds were fully characterised by a variety of techniques (e.g. SEM, DSC/TGA, FTIR, and mechanical characterisation), demonstrating excellent mechanical properties and providing sustained drug release for 4 weeks. This proof of concept study demonstrates the innovative potential of bioprinting technologies in fabrication of antibiotic scaffolds for the treatment of DFU.

**Graphical abstract:**

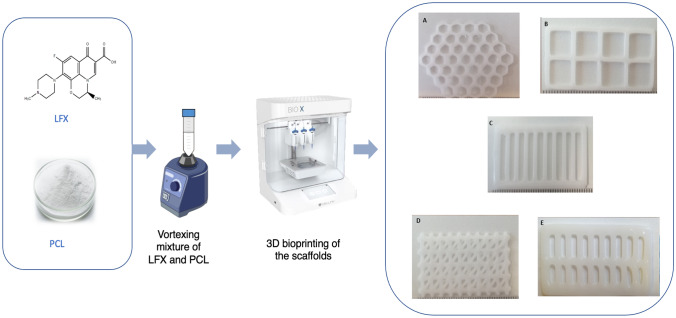

**Supplementary Information:**

The online version contains supplementary material available at 10.1007/s13346-022-01115-8.

## Introduction

DFU is a common and significant manifestation of diabetes mellitus (DM), affecting approximately one-quarter of patients [1]. The development of DFU is underpinned by several pathological factors, namely, peripheral arterial disease, a common manifestation of diabetes resulting in ischemia of the wound tissue. Furthermore, when identified, almost half of DFU are infected [2]. Standard therapies, such as pressure offloading and infection management, are often unsuccessful alone and require the introduction of advanced therapies, such as hydrogel wound dressings, which further increases treatment costs and requires hospitalisation. Biofilm formation at this stage is another major contributing factor to the estimated 70% of DFU patients who require lower-limb amputation as a result of unsuccessful treatment outcomes [1]. These outcomes have significant implications for patient quality of life, as well as increasing the costs and clinical burden in treating DFU. For further information, readers can refer to a recent review manuscript from the authors for more details on DFU pathology and treatment strategies that are commonly implemented [3].

The incidence of DM is rapidly increasing on a global scale, with an estimated 700 million people worldwide being predicted to suffer from DM by 2045 [4]. Given the rising incidence in DM, the incidence of DFU is also likely to increase in the absence of an improved and effective treatment strategy. Current market predictions forecast the global DFU market to reach $11.05 billion by 2027, an increase from a reported $7.03 billion in 2019 [5]. To overcome the economic burden involved in treating DFU, a new and innovative treatment option is required to simplify the current DFU treatment strategy and overcome restrictions faced with current treatment options.

**Fig. 1:**
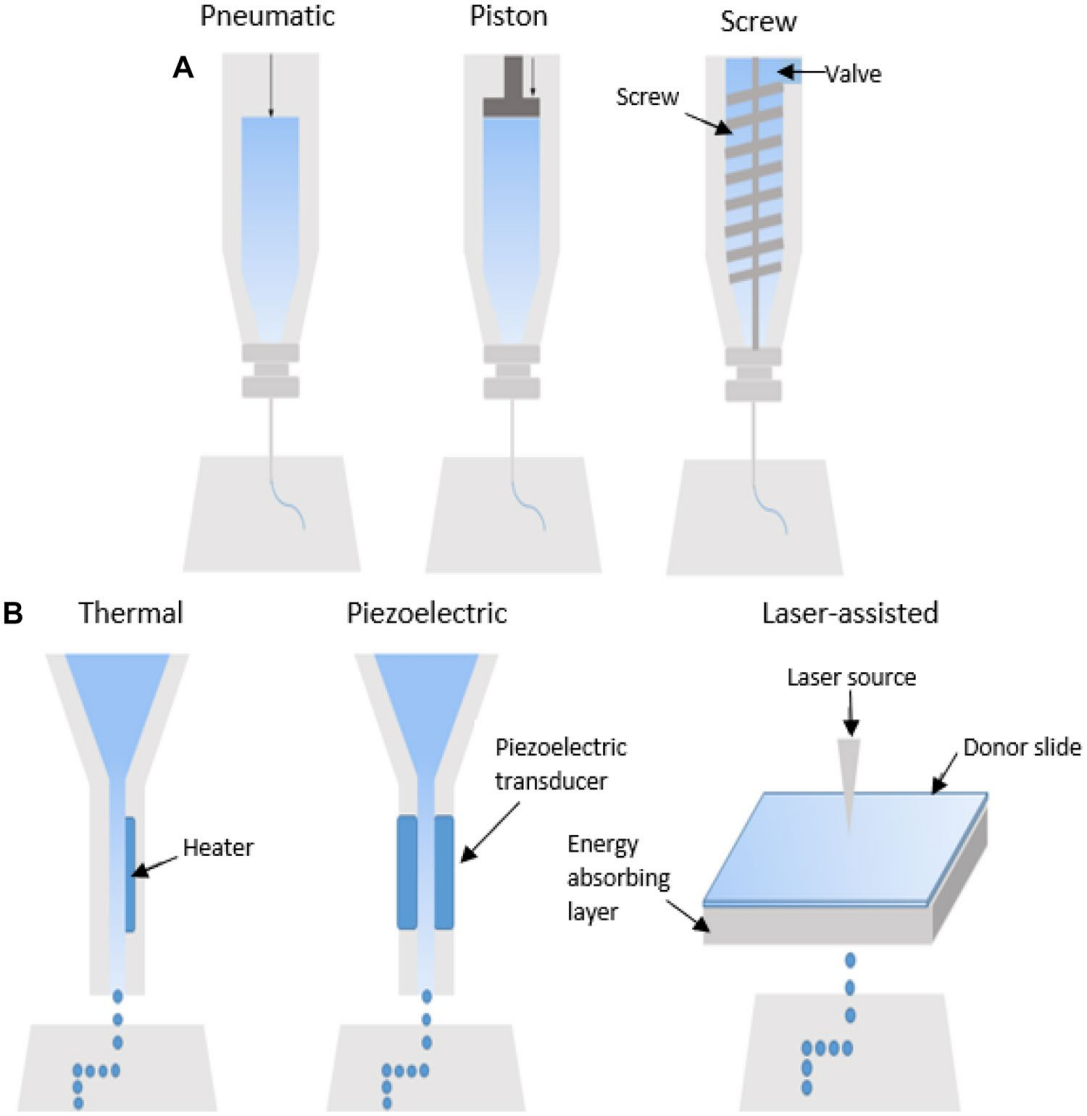
Schematics showing **a** extrusion-based, **b** inkjet-based, and **c** laser-assisted bioprinting technologies

The 3D printing method was first demonstrated in the 1980s, and since then, the field of 3D printing (3DP) and bioprinting has grown rapidly in recent years due to technological developments and greater understanding on the capabilities using these technologies, particularly in the development of scaffolds for tissue engineering applications [6]. In the fabrication of 3D (bio)printed scaffolds, the user is firstly required to design a scaffold using computer-aided design (CAD). Specialist software can then be used to slice the design prior to printing, providing a set of printing instructions for the printer; each 3DP manufacturer provides the software. A wide range of advantages are offered by 3DP, including the capability to rapidly produce elaborate (scaffold) designs of various sizes at low cost. Moreover, 3D-bioprinted scaffolds have shown the ability to achieve high cell loading and maintain high levels of cell viability [5, 7–10], while also providing structural support to the wound. The extent to which these various benefits are achieved are dependent on the chosen bioprinting technology. Currently, three main bioprinting technologies exist (Fig. 1).

Inkjet bioprinting deposits bioink droplets through piezoelectric, micro-value, or thermal mechanisms [11]. Laser-assisted bioprinting utilises an infrared source to evaporate biological materials which are coated onto a ribbon, which then are collected by the substrate as droplets [11, 12]. Extrusion-based bioprinting uses screw, pneumatic, or piston mechanisms to continuously extrude the bioink through the printer nozzle. Given the varying advantages and disadvantages of each of these technologies, as outlined in Table 1, users should carefully consider the chosen application when selecting which bioprinting technology to use. In particular, extrusion-based technologies are especially beneficial for the printing of high viscosity materials, such as polycaprolactone (PCL). PCL is commonly used for the creation of scaffolds, especially in the areas of bone and tissue regeneration. PCL is also a Food and Drug Administration (FDA)–approved material which is biocompatible, exhibiting slow degradation and good mechanical properties. Particularly for wound healing, the slow degradation of PCL can be of great benefit, as it can provide a robust supporting structure as the skin repairs over time [13].

**Table 1:** Key advantages and disadvantages of commonly used bioprinting technologies

Bioprinting technology	Advantages	Disadvantages	References
Inkjet-based bioprinting	• Low cost • High cell loading and viability • High resolutions (up to 100 µm) • Suitable for scale-up activities • Allows direct printing of cells and other biologics • Suitable for in situ bioprinting applications	• Additional processing steps may be required (e.g. chemical crosslinking) • Polymer degradation has been associated with continuous inkjet bioprinting	[7, 24–27]
Extrusion-based bioprinting	• Low cost • Higher cell seeding than inkjet-based technologies • High cell viability • Moderate (300–600 µm) to high (200 µm) resolution • Suitable for production of large scale scaffolds • Allows direct printing of cells and other biologics • Generally requires low printing temperature and pressures • Capable of printing high viscosity materials	• High temperatures may be required for high viscosity materials, ruling out the loading of biologics • Additional processing steps may be required (e.g. chemical crosslinking)	[20, 21, 28–31]
Laser-assisted bioprinting	• High-speed printing • High resolution (10 µm) • High cell loading • No nozzle required which avoids clogging issues • Suitable for in situ bioprinting purposes	• Time-consuming preparation of the ribbon for printing • More expensive than inkjet- and extrusion-based technologies • Laser source is a potential disruption to cell viability	[7, 8, 28]

Bioprinting presents an exciting opportunity to produce scaffolds for diabetic wound-healing purposes, overcoming limitations observed with alternative scaffold fabrication techniques, such as the use of volatile solvents during electrospinning processes. Many bioprinters possess more than one print head, allowing for dual extrusion of materials when printing. As bioprinting is specifically designed to allow printing with biologics, there is an opportunity to work with cells and other materials that may aid in wound healing. Other fabrication processes such as fused deposition modelling (FDM) cannot support printing with biologics, as majority of FDM printers work at too high temperatures. Bioprinters can also support the sterile manufacture of scaffolds, as printers can possess an enclosed environment for printing with the potential for UV sterilisation within the system. To allow successful DFU healing to occur, the scaffold must mimic the physiological properties of the skin. Ideally, the scaffold should provide structural support to the wound and be durable enough to withstand low-impact contact, but should also possess sufficient flexibility to adapt alongside the natural elastic nature of the skin in a normal range of body movements.

Studies have extensively shown the use of biodegradable polymers, both natural (e.g. gelatin, chitosan, collagen) and synthetic (e.g. polycaprolactone, poly(lactic-co-glycolic acid), polyethylene glycol) in nature, in the fabrication of 3D-bioprinted scaffolds for tissue engineering [14–19]. While natural polymers are typically highly biocompatible, synthetic polymers generally display more robust mechanical properties than those of natural polymers.

**Fig. 2:**
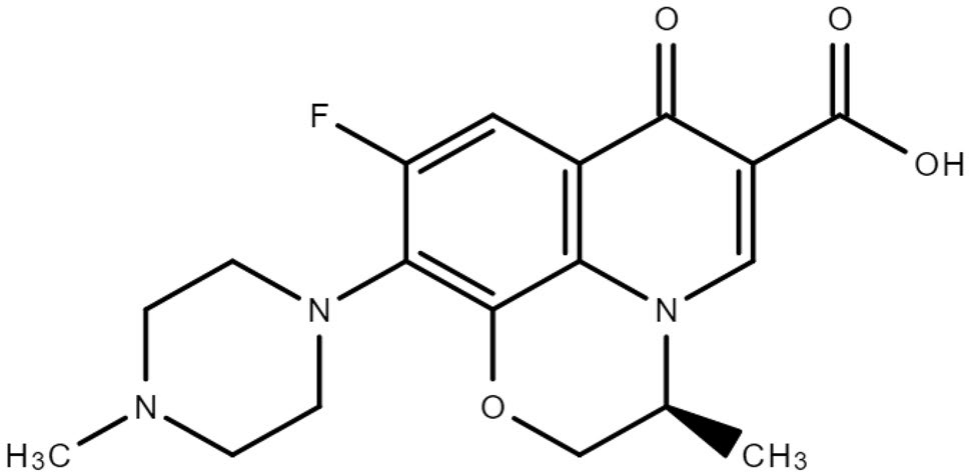
Chemical structure of levofloxacin

As outlined, nearly half of DFU become infected [2]. The presence of infection can significantly hinder the wound-healing process, leaving patients at risk of biofilm formation and lower-limb amputation. Levofloxacin (LFX) (Fig. 2) is a broad-spectrum fluoroquinolone antibiotic that has been used in the treatment of different diseases, including urinary tract infections, ocular infections, and skin infections [20]. In previous research, LFX has led to successful diabetic wound-healing outcomes when formulated in a topically-applied nanoemulsion gel, which provided infection control and acted to reduce inflammation to promote wound healing in a safe manner [21]. The use of LFX has proven beneficial in alternative applications, where it was found to successfully inhibit the growth of *Escherichia coli* and *Staphylococcus aureus*, two of the most common bacteria infecting DFU, in hernia repair and ocular applications [17, 18]. Similar results in the successful inhibition of *E. coli* and *S. aureus* by LFX have been noted in tissue engineering applications [22]. The directed delivery of antibiotics to the infection site allows to reduce the risks of toxicity related to systemic administration which require high doses to reach adequate concentrations at the infection site. Moreover, it lowers the risk of the growth of antibiotic-resistant bacterial strains [23].

In a previous study, Teo et al. used FDM to fabricate a 3-dimensional mesh with honeycomb-like pattern for the delivery of gentamicin sulphate to infected wounds [23]. In our study, the effect of the geometry of the scaffold on its mechanical properties will also be investigated, in addition to the new patterns used.

Visscher et al. prepared dual macro/microporous scaffolds by combining FDM and salt-leaching for the local delivery of the heat-labile antibiotic cefazolin [32]. First, a free-drug PCL dual macro/microporous scaffold was produced by FDM, and then, the drug was loaded using a drop-loading method. By combining these two fabrication methods, the total amount of time required to obtain the final product was quite long.

Our proof of concept study aims to develop an antibiotic-loaded scaffold using extrusion-based bioprinting technologies for the treatment of DFU.

CAD was used to produce scaffolds of various designs, which were then printed using extrusion-based bioprinting technologies. Different designs were prepared with the aim to investigate which one possesses suitable mechanical properties to provide support to the wound, while also exhibiting a degree of flexibility.

Extrusion-based bioprinting was also investigated for the purpose of directly printing LFX-loaded PCL scaffold at six different drug concentrations (0.5, 1.0, 1.5, 2.0, 2.5, and 3.0%), which were fully characterised using variety of physicochemical techniques. The antibacterial potential of the developed PCL scaffolds with LFX was assessed against relevant pathogenic bacterial strains in the context of DFU (*Staphylococcus aureus* and *Escherichia coli*).

## Materials and methods

### Materials

Polycaprolactone powder (MW 50000, PCL) was kindly provided by Ingevity (South Carolina, USA). Levofloxacin was supplied from Tokyo Chemical Industry UK Ltd. (Kasei, Tokyo, Japan). Dichloromethane was supplied from Honeywell Riedel-de Haën (Seelze, Germany). Phosphate-buffered saline (PBS, pH 7.4) tablets were purchased from Merck (Darmstadt, Germany).

*Staphylococcus aureus* NCTC 10788 (*S. aureus*) and *Escherichia coli* NCTC 10418 (*E. coli*) were maintained in cryopreservative beads in 10% glycerol at −80 °C. Both bacteria were grown in Mueller–Hinton broth (MHB) at 37 °C. For microbiology studies, Mueller–Hinton Agar (MHA) plates and Mueller Hinton Soft agar were used.

### Fabrication of control and LFX-loaded PCL scaffolds

PCL control scaffolds were fabricated using a Bio-X bioprinter thermoplastic print head (Cellink, Sweden), to which the PCL powder was added directly. Five scaffold designs were printed at a speed of 2 mm/s, under temperature and pressure parameters of 180 °C and 175 kPa, respectively. Infill density was maintained at 95% due to limitations of the bioprinter. The optimised printing parameters for PCL control scaffolds are outlined in Table 2.

**Table 2:** Optimised bioprinting parameters used to produce control PCL scaffolds

Temperature (°C)	Pressure (KPa)	Layer height (mm)	Speed (mm/s)	Infill density (%)
180	175	0.41	2	95

To prepare drug-polymer mixtures for bioprinting, 0.50% LFX was weighed out using an analytical balance and transferred to a 50-mL Falcon tube. Similarly, 99.50% PCL powder was weighed out and added to the same Falcon tube. The mixture was vortexed for 60 s to ensure homogenous mixing of the drug powder and polymer. The mixture was then transferred to a Bio-X thermoplastic print head (Cellink, Sweden) for printing. LFX-loaded scaffolds were printed at a speed of 2 mm/s, under temperature and pressure parameters of 190 °C and 175 kPa, respectively. A layer height and infill density of 0.410 mm and 100% were used, respectively. This process was repeated for five further concentrations of LFX (1.0, 1.5, 2.0, 2.5, and 3.0%).

#### Characterisation of control and LFX-loaded PCL scaffold layers

##### SEM

A TM3030 benchtop SEM (Hitachi, Tokyo, Japan) was used to evaluate the surface morphology of drug-loaded and control scaffolds at a magnification of × 50.

### Content assay

A content assay was carried out using the 3.0% LFX-loaded PCL scaffold formulation. Three scaffolds were weighed before being separately submerged in 5 mL dichloromethane (DCM) to dissolve the scaffolds. Additionally, to assess if a homogenous drug-polymer mixture had been achieved, an additional scaffold was weighed using an analytical balance before a scalpel was used to cut three small pieces from various sections of the scaffold. These sections were weighed before being submerged in 5 mL DCM to dissolve the scaffold sections. Samples were then quantified using UV–vis GENESYS™ 150 UV–Vis spectroscopy (Thermo Fisher Scientific, Massachusetts, USA) to assess the drug content and drug distribution within the scaffolds.

### FT-IR spectroscopy

Attenuated total reflection-Fourier transform infrared spectroscopy (ATR-FTIR) was used to analyse drug-loaded scaffolds (0.5–3.0%) and PCL, to conclude if any chemical interactions took place between LFX and PCL. Furthermore, pure PCL powder, printed PCL, and pure LFX powder were analysed to assess the effect of printing conditions on these substances. A spectra between 4000 and 400 cm^−1^ were obtained for each sample using a Nicolet™ iS50FTIR Spectrometer (Thermo Fisher Scientific). Samples run in triplicate, with 32 scans obtained for each sample at a resolution of 4 cm^−1^.

### TGA

Given the high-temperature printing parameters required for the bioprinting of the scaffold, a Q500 thermogravimetric analyser (TA Instruments, New Castle, DE, USA) was used to assess the thermal degradation profiles of both LFX-loaded scaffolds and control scaffolds. From 5- to 10-mg samples of each drug concentration were run in triplicate to examine weight loss. Samples were heated, at a rate of 10 °C/min, from room temperature to 500 °C. Nitrogen flow rate was maintained at 40 mL/min throughout the study. All samples run in triplicate.

### DSC

Differential scanning calorimetry (NETZSCH-Gerätebau GmbH, Wolverhampton, UK) was used to characterise the thermal properties of the drug-loaded scaffolds. Samples were weighed and placed into aluminium crucibles before being heated to 400 °C at a rate of 10 °C/min. All samples were run in triplicate to analyse the effect of LFX and printing parameters on thermal properties of the scaffold.

### Mechanical analysis

A TA.XTplus texture analyser (Stable Micro Systems, Surrey, UK) was used to assess the mechanical properties of both control and LFX-loaded scaffolds. PCL control scaffolds were printed at a size of 40 × 20 mm, using optimised parameters previously outlined in Table 2. Using a distance of 25 mm between clamps, the control scaffolds were clamped vertically into the analyser, and stretched up to 200 mm at a rate of 5 mm/s. The tensile stiffness of control scaffolds was calculated using the slope of the initial linear regression from the resulting force/displacement curves. To analyse the effect of drug-loading on scaffold mechanical properties, drug-loaded filaments for each concentration (0.5–3.0%) were produced. Using a distance of 25 mm between clamps, the filaments were clamped vertically into the analyser and stretched up to 100 mm at a rate of 5 mm/s. The slope of the initial linear regression from the resulting stress/strain graphs was used to calculate the elastic modulus. Each sample was run in triplicate.

### In vitro drug release

An in vitro release study was conducted under ambient conditions (37.5 °C), to assess and quantify LFX released from the scaffold. Scaffolds of 2 cm × 2 cm in size were printed, using conditions previously outlined, for three drug-loaded concentrations (0.5, 1.5, and 3.0%). Throughout the experiment, the scaffolds were stored in glass vials containing 10 mL of PBS. At predetermined time points, the scaffolds were removed from the glass vials, dried, and returned to the vial, with 10 mL fresh PBS being added. All samples were run in triplicate. UV–vis was used to quantify LFX at a wavelength of 292 nm, as this is the reported lambda max of LFX according to literature [33].

### In vitro antibacterial studies

Printed meshes were tested for inhibitory effects on *S. aureus* and *E. coli* bacteria. 1 cm × 1 cm squares were printed using PCL with 0.5%, 1.5%, and 3% LFX. The printed squares were sterilised by spraying with 70% ethanol and leaving to dry for approximately 3 h. One millilitre of a saturated culture of *S. aureus/E. coli* was added to a 9 ml Mueller Hinton Soft agar solution and poured onto a MHA agar plate. Once soft agar was set, the sterilised square was placed in the centre of the agar plate and left to incubate for 24 h at 37 °C. Plates were made in quintuplicate for each concentration of drug. After 24-h incubation, plates were removed and zones of inhibition recorded in mm. One plate with each bacterial type and a PCL square without drug were also made as a negative control as well as two plates with each bacterial type and no printed mesh present as a positive control. A sterility control of an MHA plate with soft agar was also made to ensure there was no contamination present.

### Statistical analysis

A one-way analysis of variance (ANOVA) was performed to detect any significant difference between the mean values of the data sets. Results were concluded to be statistically significant when *p* < 0.05. Each quantitative data set was run in triplicate and presented as a mean ± standard deviation.

## Results

### Fabrication and characterisation of control and LFX-loaded PCL scaffold

**Fig. 3:**
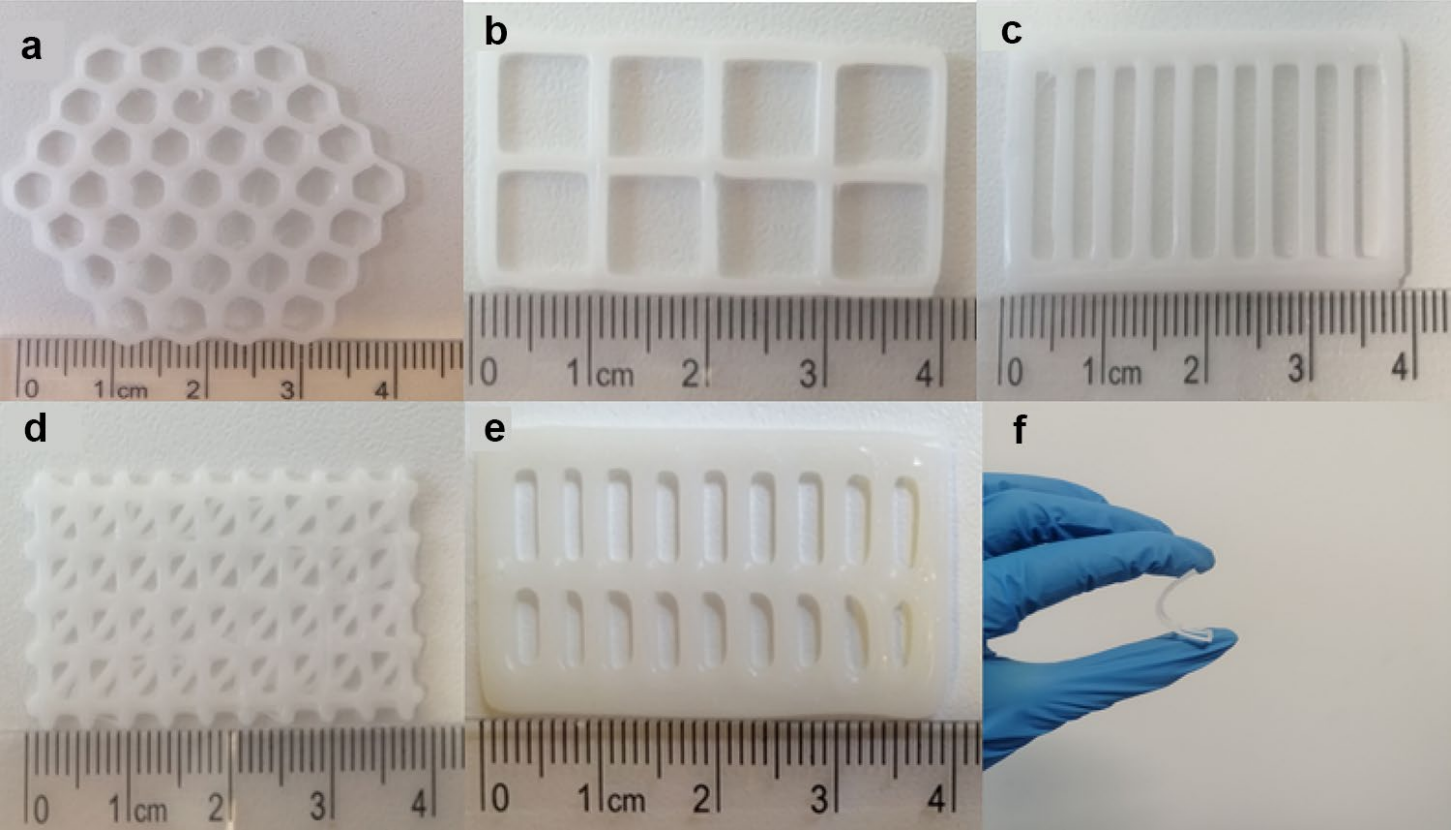
Digital images showing honeycomb (**a**), square (**b**), parallel (**c**), triangular (**d**), double-parallel (**e**), and the flexibility of a bioprinted PCL scaffold (**f**)

Five control scaffolds of various designs (Fig. 3a–e) were successfully bioprinted using PCL powder under previously outlined parameters. The LFX-loaded scaffolds of six concentrations (0.5–3.0%) were prepared by methods previously described in the sect. “Fabrication of control and LF4-loaded PCL scaffolds”. All control scaffold designs using PCL exhibited some degree of flexibility (Fig. 3f); however, mechanical properties were assessed further.

The geometry of the scaffold has effects on the resulting mechanical properties, making scaffold design rational as important as material selection. The honeycomb was selected due to previous reports of the excellent mechanical properties of this design, especially when used in combination with PCL [34]. Square and parallel designs were created with the aim to improve flexibility given that less of the total scaffold area would be composed of the PCL. The repeating unit nature of this scaffold would also allow the scaffold to be easily cut to the required size in order to reduce clinical wastage. The triangular (Fig. 3d) and double-parallel designs (Fig. 3e) were created based on the square and parallel designs respectively, to decrease the available surface area. The double-parallel design was composed by repeating units to also meet the clinical benefits outlined above.

**Fig. 4:**
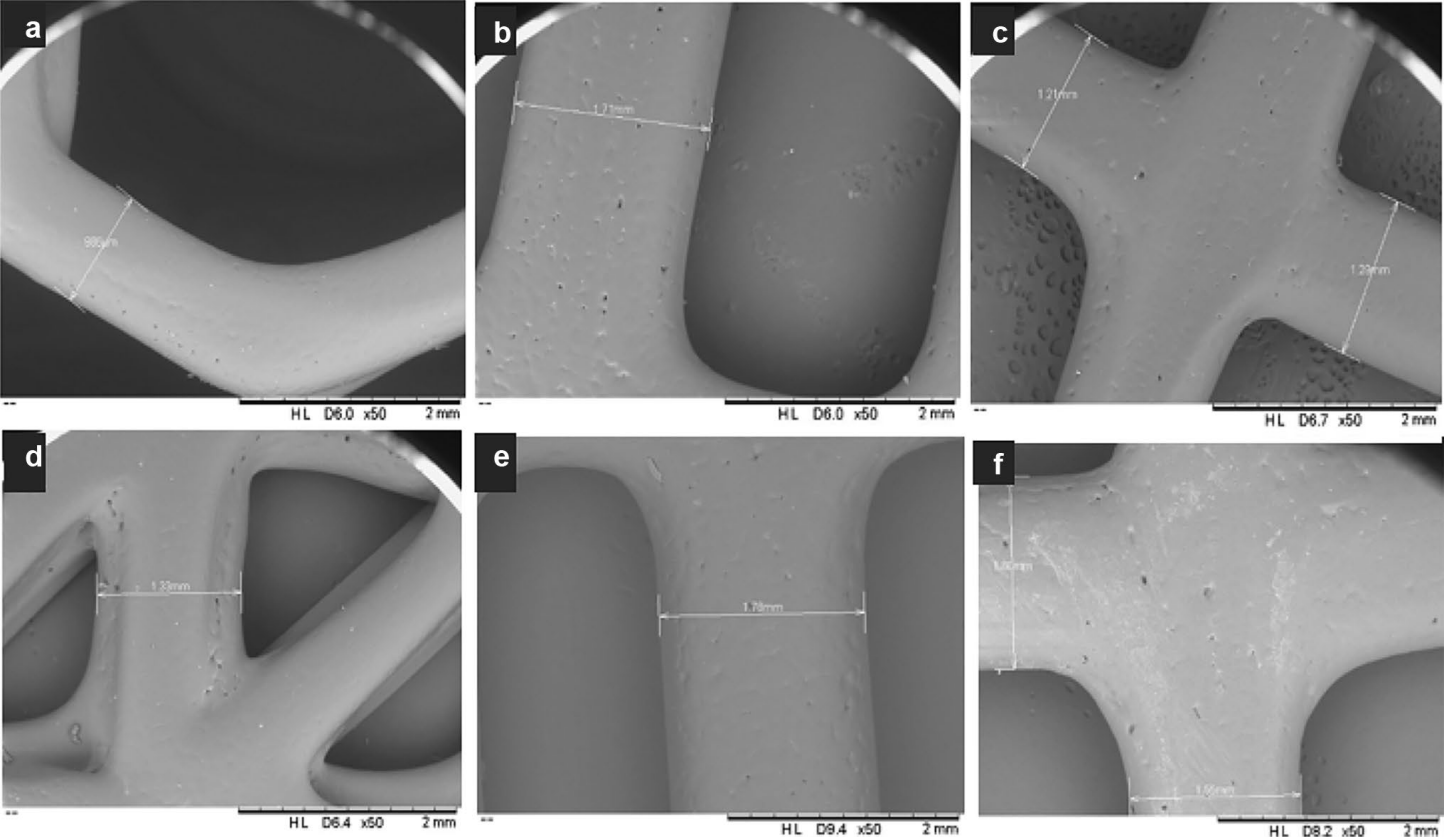
SEM micrographs of PCL control scaffolds, showing honeycomb (**a**), square (**b**), parallel (**c**), triangular (**d**), double-parallel (**e**), and a 3.0% LFX-loaded scaffold (**f**). All images were taken at × 50 magnification

To confirm that the drug powder was mixed homogenously with the PCL powder, drug distribution was assessed using the 3% LFX-loaded scaffold. Following drug quantification using UV–Vis spectroscopy, the drug was concluded to be homogeneously mixed within the scaffold following printing, and no drug had been lost during the printing process. The surface of the control scaffolds was examined using SEM (Fig. 4a–e), which appeared smooth.

**Fig. 5:**
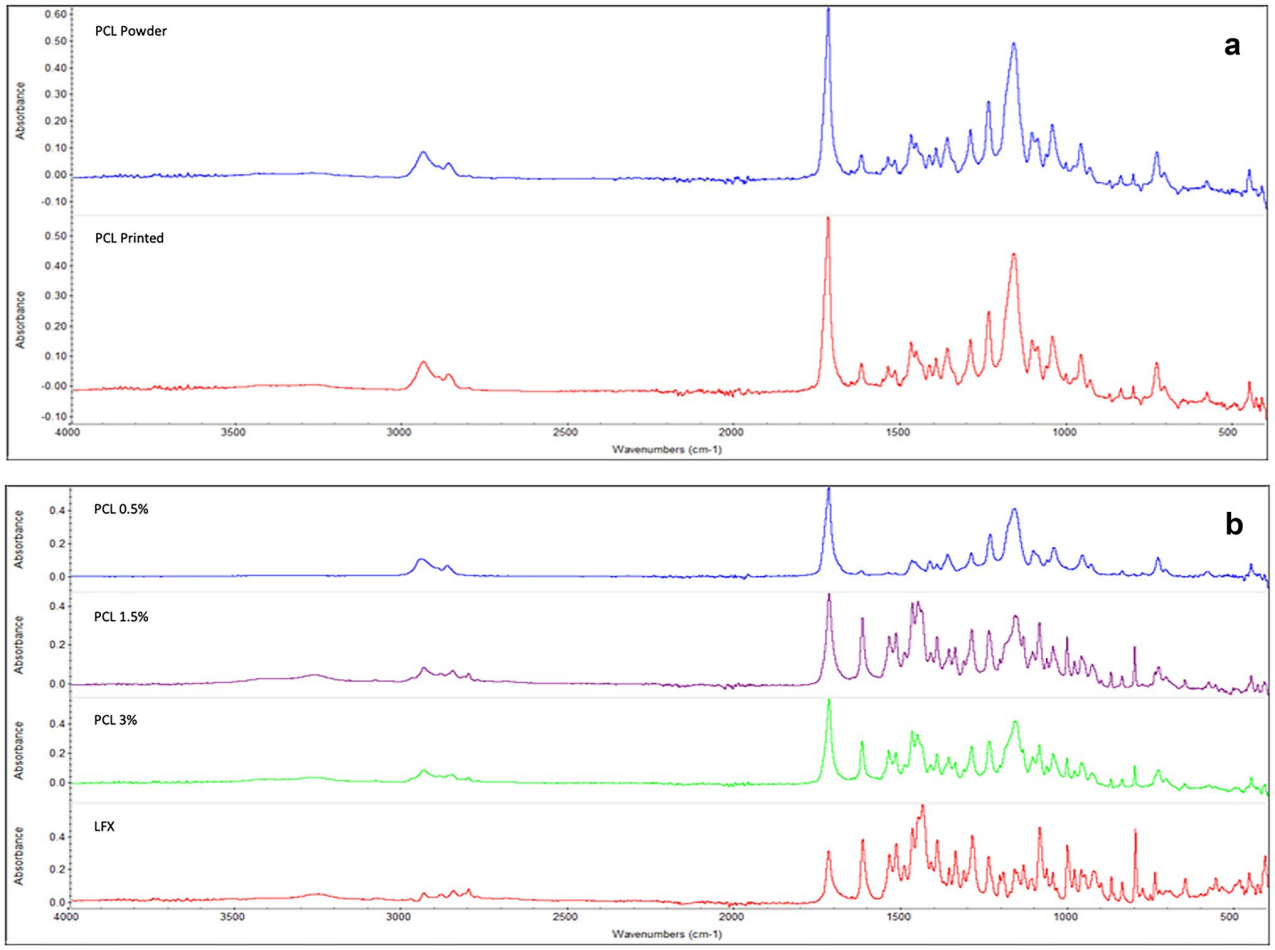
ATR-FTIR spectra showing **a** a comparison of PC powder prior to the 3D bioprinting process and printed PCL and **b** a comparison of pure LFX powder prior to the 3D bioprinting process and 0.5%, 1.5%, and 3.0% LFX-loaded formulations following printing

ATR-FTIR spectroscopy was conducted in order to identify potential interactions between LFX and PCL under the printing parameters used to fabricate the drug-loaded scaffolds. The absorption spectra obtained for the 0.5% LFX-loaded PCL scaffold (Fig. 5a, b) showed peaks highly similar to that of the pure PCL powder. The absence of a peak shift is expected to be a result of the very low concentration of drug present in this formulation. For the five remaining LFX-loaded concentrations (Fig. 5b), an additional absorption peak occurred at 3200 cm^−1^, corresponding to the hydroxyl of the carboxylic functional group within LFX. As drug concentration increased, the intensity of this peak was relatively unchanged, likely due to the small increase in drug concentrations used during this study. Peaks observed at 1700 cm^−1^ and 2900 cm^−1^ correspond to the carbonyl stretching of the carboxyl group and C–H stretching of aromatic group of LFX [35], which is further supported by findings in literature [36]. Overall, no new or unexpected peaks were identified, thus indicating FTIR did not detect any drug-polymer interaction.

For both printed and powder PCL, the peak at approximately 2950 cm^−1^ indicates the C-H stretching of alkane groups in its structure. The strong peak observed at 1700 cm^−1^ indicates C = O stretching of ester group on the PCL. The peaks obtained for both the PCL powder and the PCL sample following the printing are close to identical (Fig. 5a), indicating the printing process had no effect on PCL structure.

**Fig. 6:**
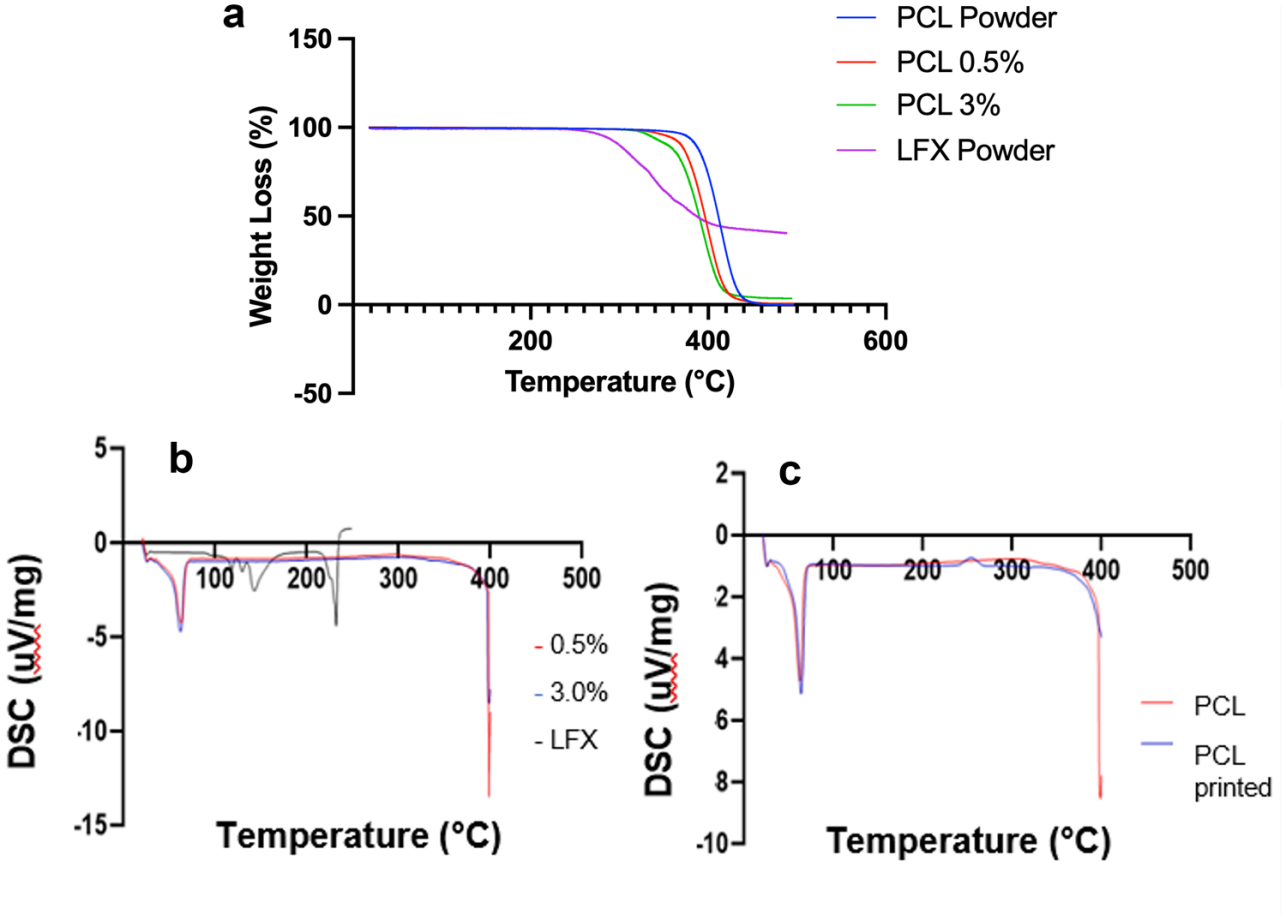
Graphs showing **a** TGA profiles of LFX powder, PCL control, and PCL loaded with 0.5% and 3.0% LFX; **b** DSC traces of LFX powder, 0.5% LFX and 3.0% LFX; and **c** DSC traces of PCL powder and printed PCL

TGA was conducted to further investigate if any interactions occurred between LFX and PCL. As shown in Fig. 6a, initial degradation occurred at higher temperatures for pure PCL powder compared to LFX-loaded samples. To assess the temperature at which thermal degradation was initiated, *T*^onset^ values were calculated. The *T*_onset_ value for PCL observed a small decline from 380 to 362 °C for both 0.5% and 3.0% LFX-loaded samples, indicating that some drug-polymer interaction had occurred; however, this effect was not further exacerbated by the range of drug concentrations used in this study. These results conclude that the addition of LFX to PCL increased the rate of thermal degradation versus control PCL.

Upon DSC analysis, LFX showed a sharp endothermic peak at approximately 235 °C (Fig. 6b) corresponding to its melting point, which is supported by literature [20, 24]. Similarly, LFX showed a *Tg* of 27 °C. PCL showed a glass transition temperature of 27 °C (Fig. 6c). Furthermore, a sharp endothermic peak appeared at 62 °C, corresponding to the expected melting point of PCL [37].

### Mechanical properties of control and drug-loaded scaffolds

The effect of scaffold geometry on the resulting mechanical properties was analysed using texture analysis. Ideally, the scaffold should provide structural support to the wound and be resistant to low-impact force. The scaffold should exhibit some degree of flexibility in order to respond to the natural stretching and movement of the skin during a normal range of body motions.

Bioprinting technologies have shown the ability to produce flexible scaffolds, which still retain desirable mechanical characteristics, unlike techniques such as fused deposition modelling (FDM) 3D printing, which can produce very rigid scaffolds unsuitable for tissue engineering applications [38].

**Fig. 7:**
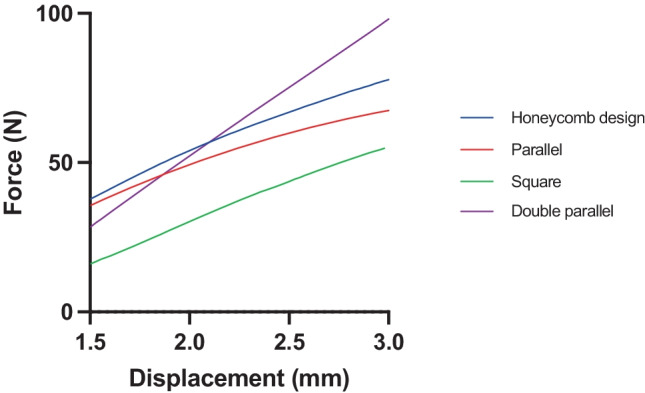
Force/displacement graphs for honeycomb design, parallel, square, and double-parallel control designs

As shown in Fig. 3f, all control scaffolds demonstrated some degree of flexibility. The mean tensile stiffness of each layer design was calculated using the slope of the initial linear section of the corresponding force/displacement curves. The triangular design was not suitable for analysis due to limitations of the texture analyser. This design was very rigid and could not be gripped properly by the clamps during tension in order to get an accurate measurement; therefore, it was omitted from analysis. As shown in Table 3, the square design showed the lowest mean tensile stiffness of 25.30 N/mm, meaning this scaffold would demonstrate the most appropriate level of flexibility for its application. While the double-parallel design showed the greatest tensile strength, indicating greater force is necessary for elongation during the initial elastic region, these findings were not significantly different (*p* > 0.05) to values obtained for the remaining scaffold layer designs. Figure 7 shows the force/displacement graphs obtained for both square and double-parallel designs. Given the desirable mechanical properties observed, the square design was selected for further analysis.

**Table 3:** Tensile stiffness of PCL scaffold layers under optimised printing parameters

Mesh design	Tensile stiffness (N/mm)
Honeycomb	28.57 ± 8.0
Square	25.30 ± 2.9
Parallel	26.44 ± 12.2
Triangular	-
Double-parallel	48.87 ± 2.7

**Fig. 8:**
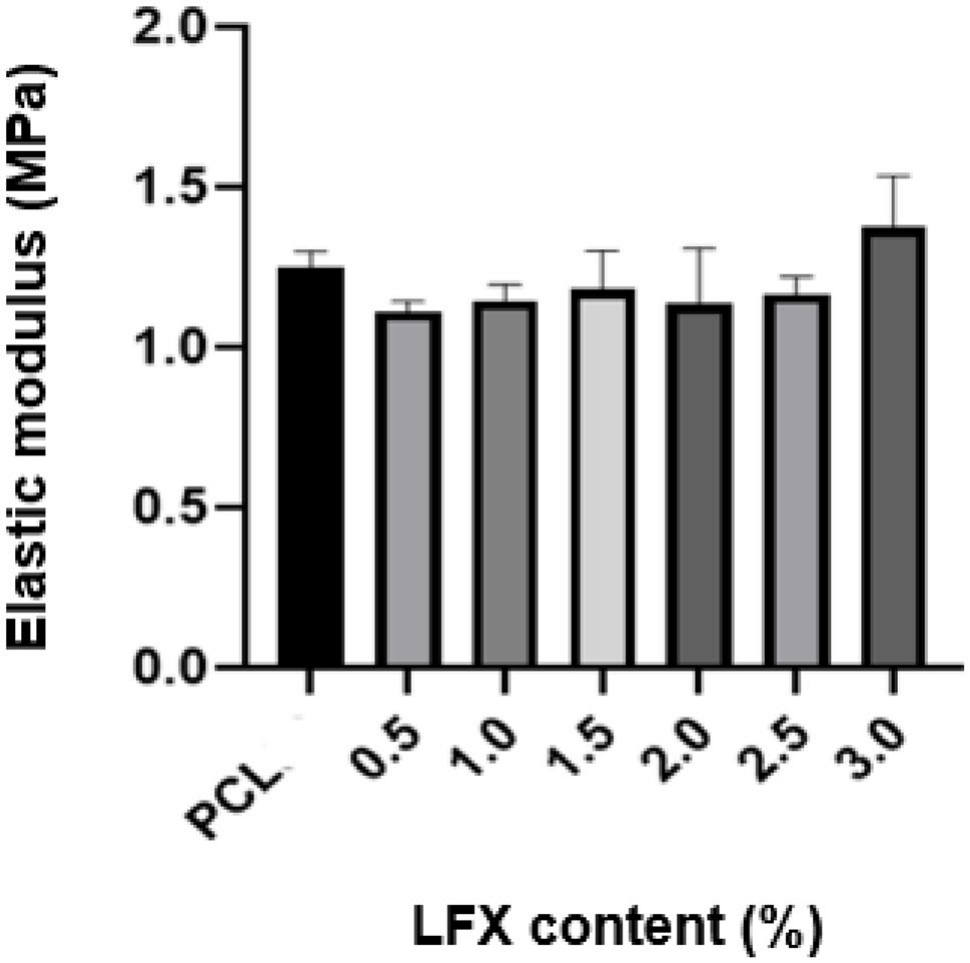
Graph showing the effect of the percentage LFX loading into PCL scaffold on their elastic modulus

To determine the effect of drug-loading on the mechanical properties of the scaffolds, the elastic modulus was calculated for the 6 drug-loaded filaments at a concentration of 0.5–3.0%. Higher elastic modulus values indicate the material to be stiff, making it highly resistant to elastic deformation when subject to stress [39]. As shown in Fig. 8, the presence of LFX had no significant effect on the elastic modulus of PCL (*p* > 0.05). Furthermore, the filaments demonstrated elasticity during this experiment, as none fractured under the testing conditions used. Given these results, in order to assess the full range of drug-loaded scaffolds, 0.5%, 1.5%, and 3.0% LFX PCL scaffolds were selected for in vitro drug release analysis using square design.

### In vitro drug release

**Fig. 9:**
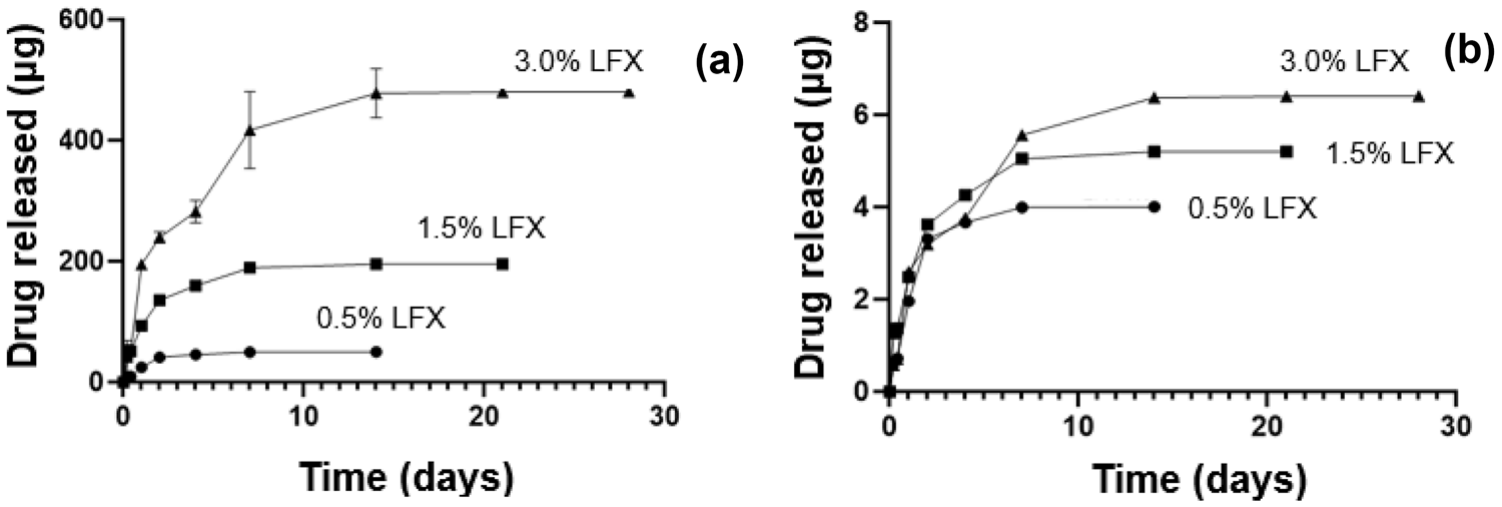
Graph showing (**a**) cumulative in vitro drug release profile of LFX from drug-loaded scaffolds of varying concentrations (*n* = 3) and (**b**) percentage drug release profile of LFX from drug-loaded scaffolds with varying LFX loadings (*n* = 3)

In vitro drug release analysis was conducted using 0.5%, 1.5%, and 3.0% drug-loaded formulations of the square design in order to assess the full range of drug-loaded formulations used in this study. All formulations showed an initial burst release phase followed by steady-state release. At day 14, there was a significant increase in the quantity of LFX released for the 0.5% formulation (50.13 µg) and 3.0% formulation (478.23 µg) (Fig. 9). Moreover, the 3.0% formulation also showed a higher percentage release, based on initial drug-loading quantities, at day 14 (6.37%) versus the 0.5% formulation (4.01%) (Fig. 9b). As the percentage drug release increases with increasing drug concentration, it can be assumed that there is no major drug-polymer interaction impeding drug release the matrix. Steady-state drug release was achieved following day 4, 7, and 14 for 0.5%, 1.5%, and 3.0% LFX, respectively. The 3.0% LFX provided the longest duration of release, indicating its suitability for sustained drug delivery of the antibiotic. The different duration of the release studies reported in Fig. 9 was due to the time needed to reach the steady state being different for different concentrations; the scaffolds with the lowest concentration achieved before the steady state—experiments stopped once the steady state was achieved.

### In vitro antibacterial studies

In vitro antibacterial studies were conducted using 0.5% and 1.5% drug-loaded formulations of the square design in order to assess the inhibitory effects on *S. aureus* and *E. coli* bacteria. We used as negative control a PCL square without LFX and as positive control two plates with each bacterial type and no printed scaffold. As expected, the control scaffolds containing no LFX had no zones of inhibition in both bacteria (Figs. S1 and S2).

**Fig. 10:**
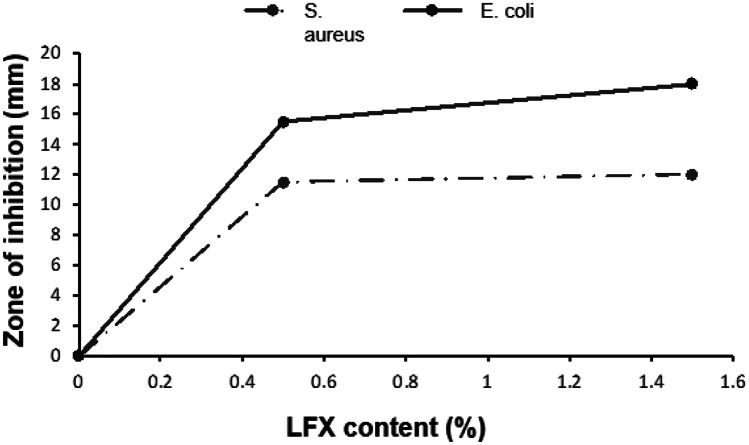
Diameter of the zone of inhibition of *S. aureus* and *E. coli* with 0.5% and 1.5% levofloxacin-loaded scaffolds

As shown in Fig. 10, there was no major difference between the zone of inhibition of *S. aureus* determined at the two different LFX concentrations (0.5% and 1.5%). Therefore, it can be inferred that an increase in the LFX concentration from 0.5 to 1.5% did not have a significant impact on the zones of inhibition produced. The same trend was observed in the inhibition zones of *E. coli*. However, the diameter of the zones of inhibition of *E. coli* was higher than the produced in *S. aureus.* Therefore, LFX-loaded scaffolds possess a higher inhibitory effect on *E. coli* than on *S. aureus.* This is in accordance with the studies of Lemmen et al. who investigated the bactericidal activity of moxifloxacin and levofloxacin against *Staphylococcus aureus*, *Staphylococcus epidermidis*, *Escherichia coli*, and *Klebsiella pneumoniae*. In this study, the minimal inhibitory concentration (MIC) and the minimal bactericidal concentration of LFX were higher for *S. aureus* than for *E. coli* [40].

## Discussion

Bioprinting is a technology, which is gaining increasing attention within the field of tissue engineering. One advantage of bioprinting is the wide variety of materials suitable for bioprinting, namely biodegradable polymers. Bioprinting is capable of producing composite scaffolds, composed of both synthetic and natural polymers, to combine the benefits of each polymer type and alleviate the respective limitations.

Additive manufacturing (AM) technologies, such as FDM, and electrospinning have been used extensively in the production of scaffolds for tissue engineering purposes; however, these methods incur limitations. Electrospinning, for example, often requires the use of highly volatile solvents during processing, while FDM has been known to produce highly rigid scaffolds which are unsuitable for tissue engineering purposes [38]. Bioprinting technologies overcome these limitations with the ability to rapidly produce scaffolds of various geometries and sizes without the need of volatile solvents. Furthermore, these scaffolds are durable and have suitable mechanical properties to provide support to the wound, while also exhibiting a degree of flexibility. Bioprinting also offers the opportunity to print bioinks containing cells which often display high viability [5, 9], notably for natural biodegradable polymers, which require low processing temperatures.

PCL has been used in a number of studies for tissue engineering purposes where it has displayed excellent mechanical properties, alongside desirable biocompatibility [41]. Given this, it was selected for this study to fabricate the scaffold, providing the structural framework of the scaffold.

Many factors contribute to the impaired wound-healing response, such as the presence of diabetic neuropathy and peripheral arterial disease as manifestations of diabetes [42, 43]. Infection, particularly biofilm formation witnessed in the later stages of DFU progression is another major contributing factor to poor wound-healing outcomes in diabetic patients. For mild infection, antibiotics are often administered orally, which limit the bioavailability due to the first-pass metabolism, as well as requiring higher doses, which can risk inducing toxicity. While intravenous administration of antibiotics, often used for more severe infection, bypasses the first-pass metabolism experienced during oral administration, this route is invasive, requires hospital treatment and chronic administration via this route is not feasible. Therefore, topical administration of antibiotics can be used as an alternative method to provide sustained, localised drug delivery to the target site, with low potential for systemic side effects and ease of administration being additional benefits of this route.

LFX, a fluoroquinolone antibiotic, was selected for incorporation into the scaffold. Levofloxacin has been successful in many wound-healing applications [16, 20]. Previous studies have used levofloxacin concentrations at a range of 0.5–1.5% for tissue engineering applications, with similar values observed for topical administration to the eye [44, 45]. LFX has been used extensively in the treatment of ocular infection. Studies investigating the efficacy of topical LFX applied to the cornea found 3.0% LFX to cause ocular damage and therefore be unsafe for use in terms of ocular drug delivery [46]. Despite this, the bactericidal ability of LFX up to concentrations of 3% was investigated. The main role of the skin is to provide protection given its multilayer nature; thus, it is less sensitive to higher drug concentrations that the eye, which is more sensitive to toxicity.

Lower concentrations of 0.5–1.5% have been widely investigated and proven beneficial in the topical applications of LFX in eradicating commonly exposed bacteria such as *S. aureus* and *E. coli* [19, 44, 45]; therefore, in this study, higher concentrations were also used in order to investigate any potential benefits.

During this proof of concept study, scaffolds with different designs, loaded with antibiotic were fabricated using bioprinting technologies. The temperature for printing was increased for LFX-loaded scaffolds to accommodate the drug loading. To fabricate the scaffolds, PCL loaded with LFX was successfully printed using optimised parameters for extrusion-based bioprinting before being characterised. Due to the high viscosity of PCL, slower printing speed was required to maintain shape fidelity of the scaffold. Following analysis of drug distribution confirmed that even drug distribution was achieved.

Both control and LFX-loaded scaffold were observed using SEM and presented smooth surfaces. Importantly, the LFX-loaded scaffolds did not show any signs of drug aggregation on the surface of the scaffold.

During ATR-FTIR analysis, no peak shift was observed for the lowest concentration used in this study (0.5%), suggesting this concentration was too low to acquire any conclusive ATR-FTIR result. Thermal analysis using TGA was subsequently performed to assess any interactions occurred between PCL and LFX. It was found that PCL displayed a higher *T*_onset_ value of 380 °C, which saw a slight decline to 362 °C for both 0.5% and 3.0% LFX-loaded samples. Despite the incorporation of LFX causing a slight decrease in the observed thermal resistance for PCL, the temperatures used during the 3D bioprinting process were lower than the degradation temperatures, concluding that 3D bioprinting is suitable for the fabrication of LFX-loaded PCL scaffolds. Similar reports for PCL and LFX combinations have been reported in literature [47]. DSC analysis was also conducted to assess thermal properties of the components of the scaffold. A strong endothermic peak was observed at approximately 235 °C for LFX, which corresponds to its melting point and is supported by literature [22, 24]. For the drug-loaded scaffolds, this sharp peak disappeared. This could result from dilution of the drug compared to the amount of polymer used in these formulations, or as a result of drug amorphisation during the printing process*.* The sharp endothermic peak observed for PCL occurred at 62 °C, corresponding to the expected melting point of PCL [37]. Both LFX and PCL showed a low *Tg* temperature, which is desirable for ease of processing.

During this study, it was found that modifying the geometry and design of the scaffold, resulted in a change to the mechanical properties displayed. While the scaffold should provide support to the wound, it must also exhibit some degree of flexibility in order to respond to the dynamic nature of the skin in a normal range of body movements. In this study, the square design displayed the lowest tensile stiffness and therefore was selected for in vitro drug release analysis. The triangular design was not suitable for analysis due to limitations of the texture analyser, due to reasons previously outlined, and therefore was omitted from this experiment.

In clinical practice, the scaffold could be applied to the skin using an adherent dressing that would aid the retention of the scaffold in direct contact with the skin over the desired application time. Such bioadhesives have been fabricated from a number of materials, including polyethylene glycol and chitosan to aid wound healing [48]. Other commonly used bioadhesives for wound closure, such as cyanoacrylate-based bioadhesives, have however been shown to be unsuitable for application to areas vulnerable to torsion or moist tissues [48]; therefore, these factors should be considered when selecting an appropriate bioadhesive material to aid scaffold retention. Furthermore, a gauze and bandage could also be used to further secure the positioning of the scaffold and could be easily removed at time points where the scaffold was required to be replaced. These options would offer greater freedom of mobility for the patient versus total contact casts commonly employed in the treatment of DFU.

Insight into the effect of drug concentration on the mechanical properties of the scaffolds was also conducted. Given that there was no significant difference in the elastic modulus values obtained for PCL and drug-loaded PCL samples, it can be concluded that the addition of LFX had no effect on the elastic modulus of the first scaffold layer. Given this, concentrations of 0.5, 1.5, and 3.0% LFX-loaded scaffold were selected for in vitro drug release to observe the release over the range of drug concentrations. The results showed significantly lower elastic modulus results than reported in similar studies using PCL and LFX to produce electrospun scaffolds [47]; however, values obtained are closer to the previously reported elastic modulus values (1.14 MPa) of plantar soft tissue [33, 34].

During the in vitro drug release analysis, all the concentrations (e.g. 0.5%, 1.5%, and 3.0%), demonstrated similar release profiles, with an initial burst release phase followed by steady-state release. Despite a burst release being observed, it was lower than previous studies investigating the release of LFX from PCL electrospun scaffolds, where 50% of drug had been released by 12 h [45]. Moreover, the 3.0% formulation also showed a higher percentage release, based on initial drug-loading quantities, at day 14 (6.4%) versus the 0.5% formulation (4.0%). Given that increasing the drug-loaded concentrations results in increased drug release can be concluded that no major drug-polymer interaction occurred. Steady-state drug release was achieved following day 4, 7, and 14 for 0.5%, 1.5%, and 3.0% LFX, respectively. The 3.0% LFX provided the longest duration of release, indicating its suitability for sustained drug delivery of the antibiotic. The low drug release values obtained for these formulations can be owed to the slow hydrolytic degradation of PCL which is required to release the drug from the polymer matrix [49]. Furthermore, this avoids potential toxicity, which can result from high burst release. If required, the rate of drug release can be increased through changing the PCL molecular weight or through structure modification using hydrophilic moieties.

## Conclusions

This proof of concept study outlines the capabilities of bioprinting technologies to produce scaffolds able to provide sustained release of antibiotic to infected DFU. In this study, the chosen scaffold design demonstrated suitable mechanical properties for tissue engineering purposes and can be easily modified to the size of the wound. Moreover, the study has provided a simplified, low-cost alternative to current DFU treatments, which are often unsuccessful and incur significant costs, thus demonstrating the potential of bioprinting to revolutionise DFU treatment. In future directions, it would be interesting to include in the scaffold a further layer with the purpose to provide dual administration of therapeutics through inclusion of drugs, cells, or biologics. Overall, this approach would act to simplify the current DFU treatment strategy, thus reducing the economic burden caused by rapidly increasing patient demand.

## Supplementary Information

Below is the link to the electronic supplementary material.

Supplementary file1 (DOCX 2.53 MB)

## Data Availability

The datasets generated during and/or analysed during the current study are available from the corresponding author on reasonable request.
