# Supplementary material for: 3D bioprinted scaffolds for diabetic wound-healing applications
Source: Drug Deliv Transl Res. 2022 Jan 11;13(8):2096–109. doi: 10.1007/s13346-022-01115-8 (PMC10315349; doi:10.1007/s13346-022-01115-8)
Supplement: Supplementary file 1 — Supplementary file1 (DOCX 2.53 MB) [file 13346_2022_1115_MOESM1_ESM.docx]

3D bioprinted scaffolds for diabetic wound healing applications

Katie Glover, Essyrose Mathew, Giulia Pitzanti, Erin Magee, Dimitrios A. Lamprou*

School of Pharmacy, Queen’s University Belfast, 97 Lisburn Road, Belfast BT9 7BL, UK

Corresponding Author

Dimitrios A. Lamprou

E-mail: [d.lamprou@qub.ac.uk](mailto:d.lamprou@qub.ac.uk)


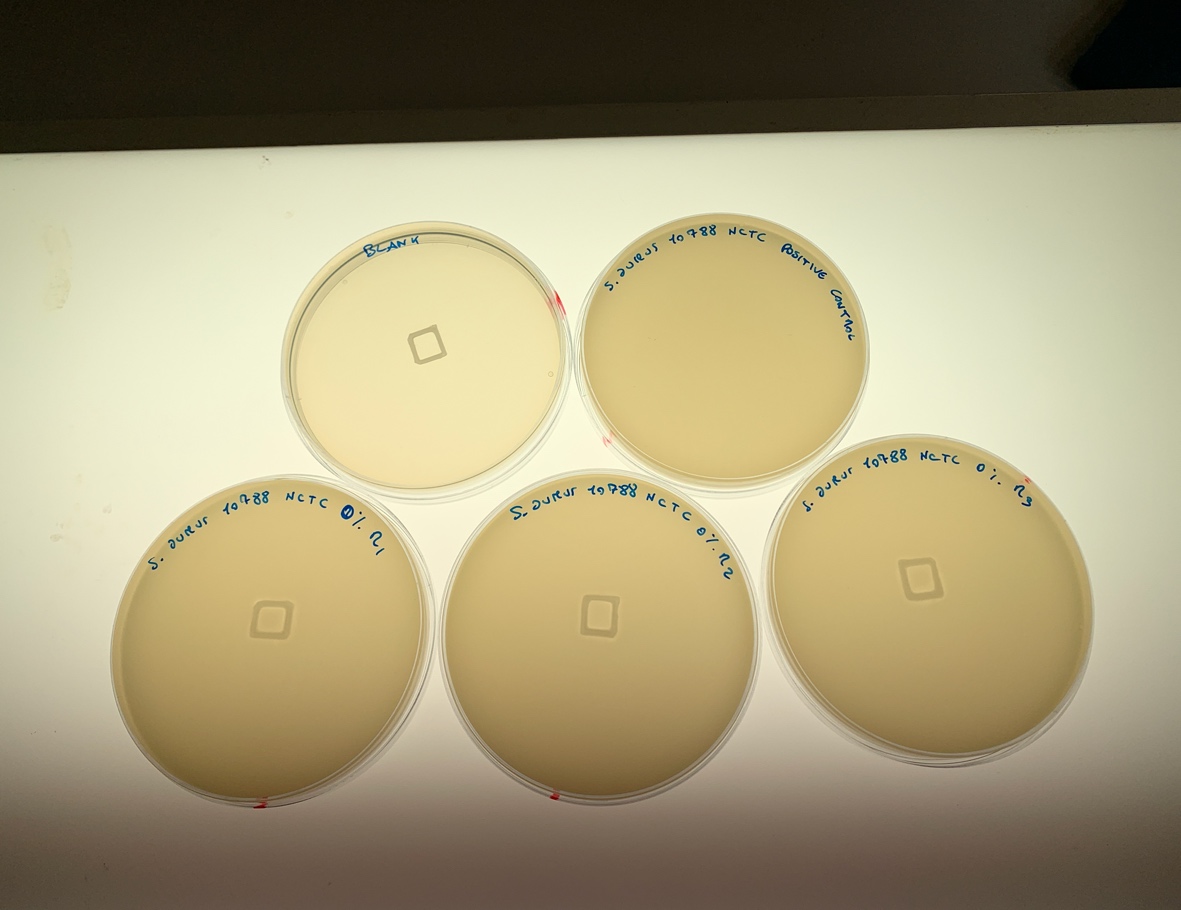


b

aa

e

d

c

**Fig. S1** Blank (a), positive control (b) and inhibitory activity of unloaded scaffolds on S.aureus (c-e).


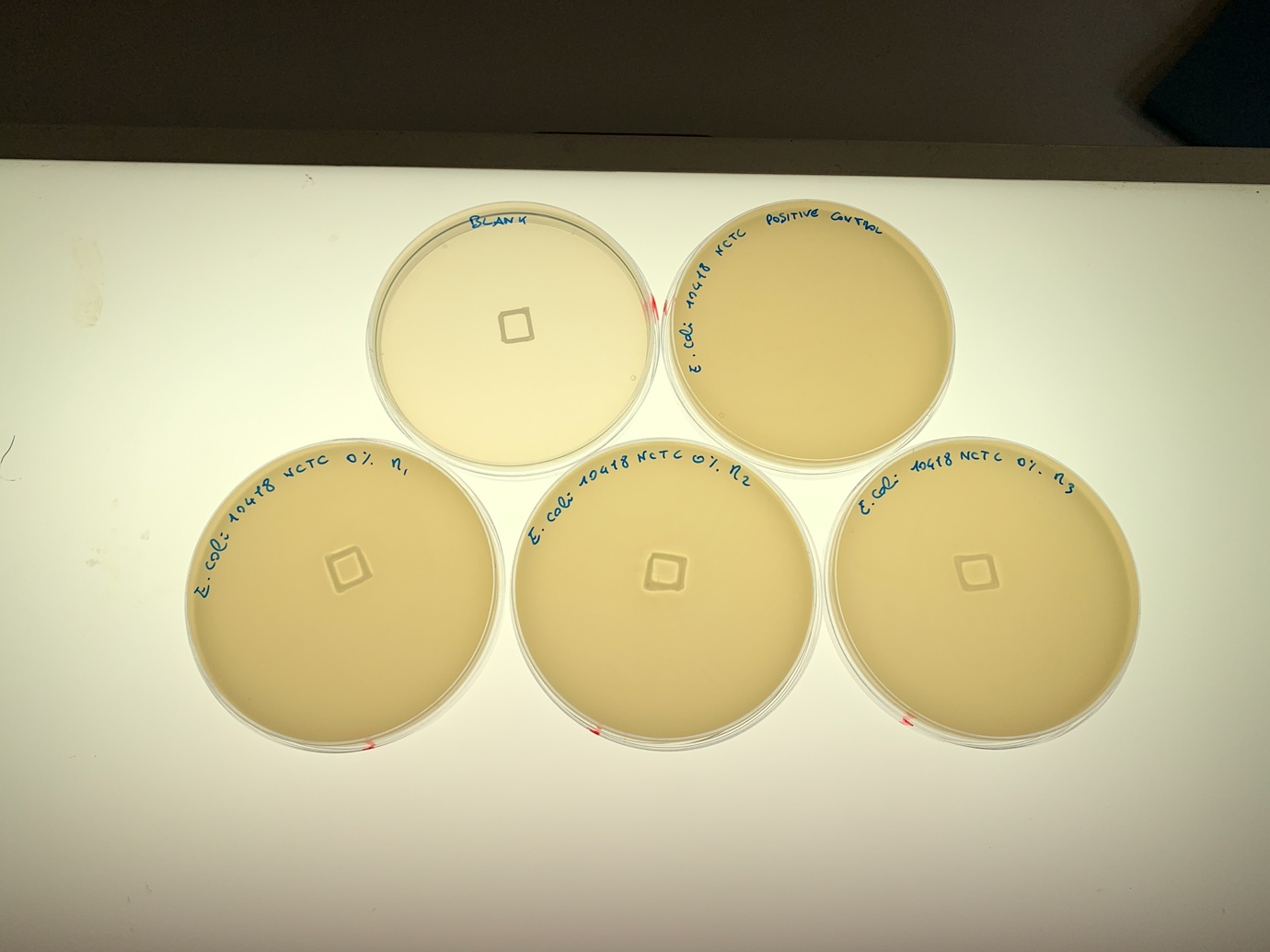


c

d

e

a

b

**Fig. S2** Blank (a), positive control (b) and inhibitory activity of unloaded scaffolds on E.coli (c-e).


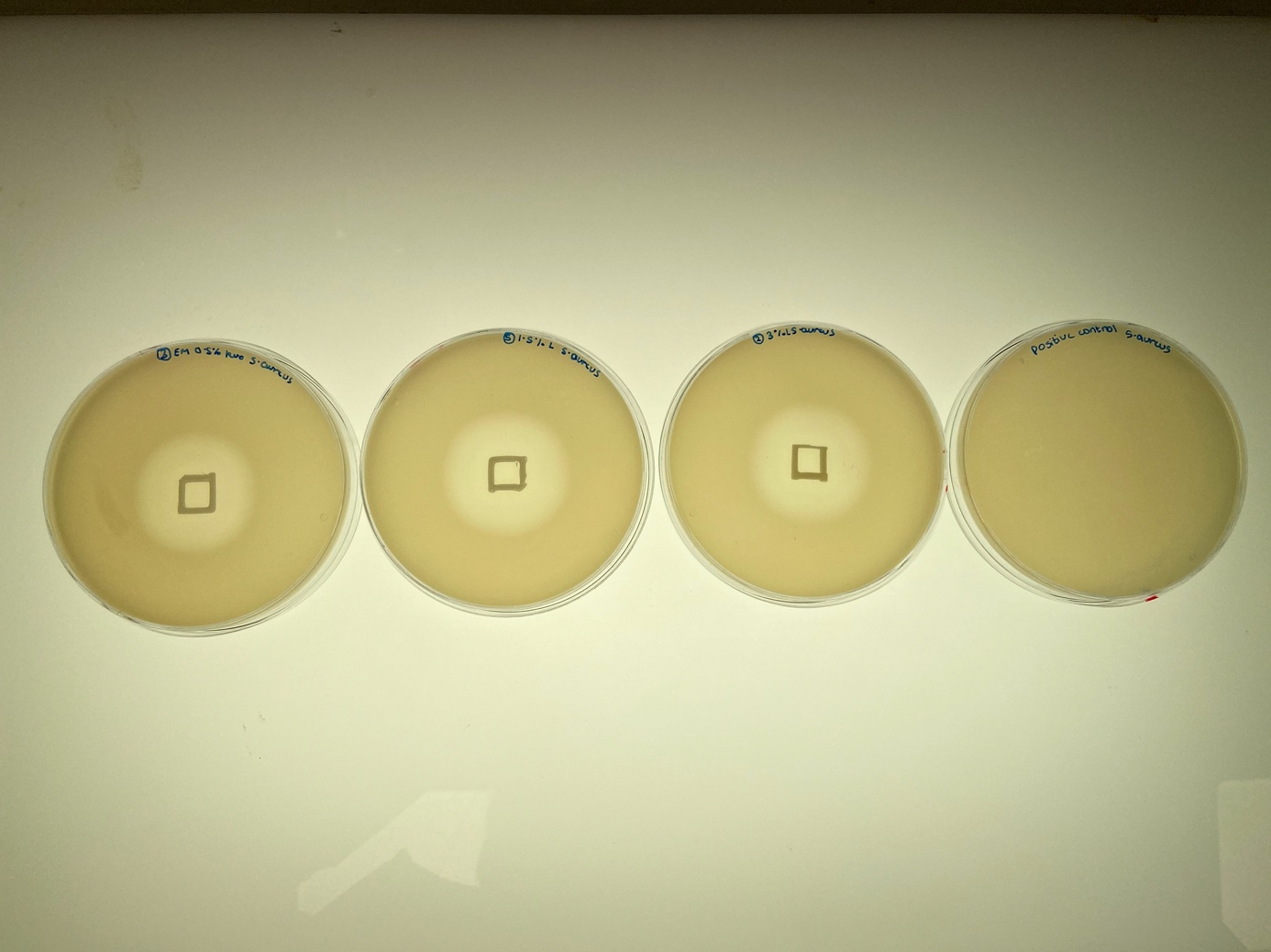

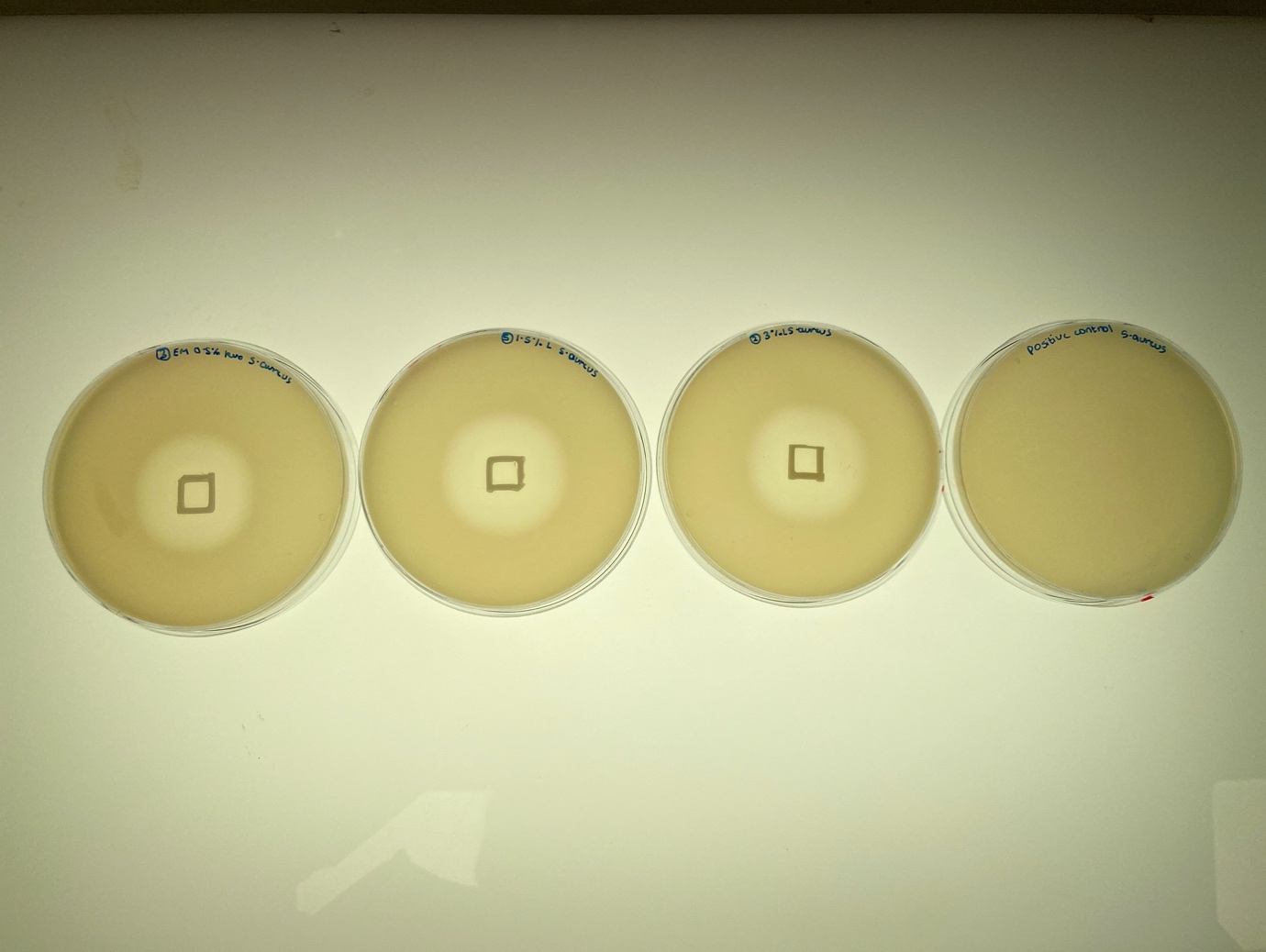


a

b

c

**Fig. S3** Inhibitory activity of 0.5% (a) and 1.5% (b) LFX loaded scaffold on S.aureus and positive control (c).


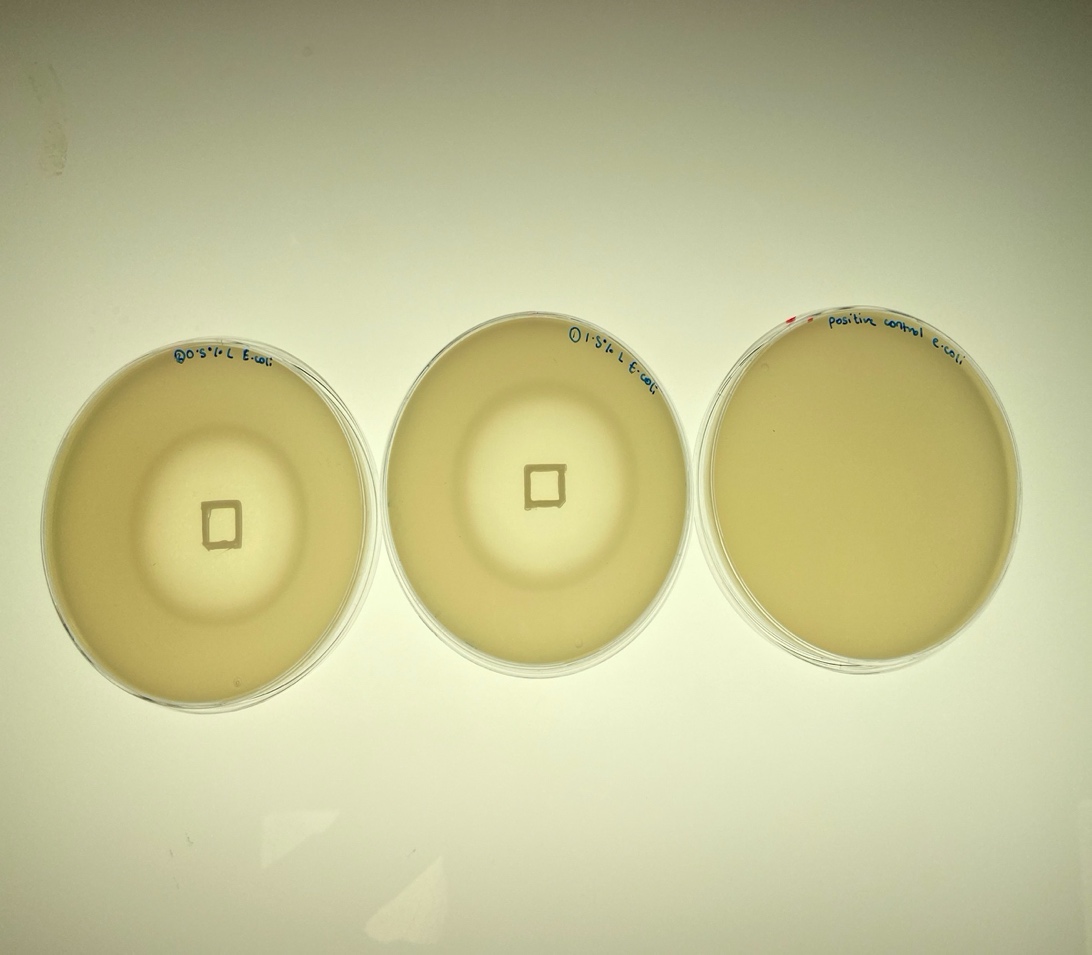


a

bBb

cBc

**Fig. S4** Inhibitory activity of 0.5% (a) and 1.5% (b) LFX loaded scaffold on E.coli and positive control (c).
